# Dynamic microtubule organization and mitochondrial transport are regulated by distinct Kinesin-1 pathways

**DOI:** 10.1242/bio.015206

**Published:** 2015-11-18

**Authors:** Anna Melkov, Yasmin Simchoni, Yehonatan Alcalay, Uri Abdu

**Affiliations:** Department of Life Sciences, Ben-Gurion University, Beer-Sheva 8410500, Israel

**Keywords:** Bristle, Drosophila, Kinesin, Microtubule, Mitochondria

## Abstract

The microtubule (MT) plus-end motor kinesin heavy chain (Khc) is well known for its role in long distance cargo transport. Recent evidence showed that Khc is also required for the organization of the cellular MT network by mediating MT sliding. We found that mutations in *Khc* and the gene of its adaptor protein, kinesin light chain (Klc) resulted in identical bristle morphology defects, with the upper part of the bristle being thinner and flatter than normal and failing to taper towards the bristle tip. We demonstrate that bristle mitochondria transport requires Khc but not Klc as a competing force to dynein heavy chain (Dhc). Surprisingly, we demonstrate for the first time that Dhc is the primary motor for both anterograde and retrograde fast mitochondria transport. We found that the upper part of *Khc* and *Klc* mutant bristles lacked stable MTs. When following dynamic MT polymerization via the use of GFP-tagged end-binding protein 1 (EB1), it was noted that at *Khc* and *Klc* mutant bristle tips, dynamic MTs significantly deviated from the bristle parallel growth axis, relative to wild-type bristles. We also observed that GFP-EB1 failed to concentrate as a focus at the tip of *Khc* and *Klc* mutant bristles. We propose that the failure of bristle tapering is due to defects in directing dynamic MTs at the growing tip. Thus, we reveal a new function for Khc and Klc in directing dynamic MTs during polarized cell growth. Moreover, we also demonstrate a novel mode of coordination in mitochondrial transport between Khc and Dhc.

## Abstract (precis)

**Summary:** Highly polarized *Drosophila* bristle cells reveal that dynamic microtubule organization and mitochondrial transport are regulated by distinct Kinesin-1 pathways, and a novel mode of coordination between Khc and Dhc in mitochondrial transport.

## INTRODUCTION

In interphase cells, long distance transport along microtubules (MTs) is driven by motor proteins. Whereas the cytoplasmic dynein complex selectively transports cargo towards the MT minus end, most kinesins carry cargo towards the MT plus end. Kinesin-1 is a heterotetrameric motor protein composed of two 115 kDa heavy chains (Khc), which are responsible for motor activity, and two 58 kDa light chains (Klc) that serve as adaptor proteins. Apart from Klc, other adaptors and scaffold proteins were found to regulate Khc. Some of these adaptors, such as the *Drosophila* Sunday driver (Syd) and its mammalian homologues JIP3/JSAP1, require Klc for regulation of Khc-dependent cargo transport (Bowman et al., 2000). Other adaptors, such as Milton, which regulates Khc-mediated mitochondria transport in axons, act in a Klc-independent manner (Glater et al., 2006). In addition to its involvement in cargo transport, accumulating evidence points to a role for Khc in regulating other cellular processes. One such process is the organization of the cellular MT network. For instance, Khc is required, independently of Klc, for MT sliding in mammalian and *Drosophila* cells (Jolly et al., 2010; Metzger et al., 2012), as well as for initial neurite outgrowth in *Drosophila* neurons (Lu et al., 2013). Moreover, Khc is also involved in regulating dendrite MT polarity in *C. elegans* (Yan et al., 2013)*.*

To better understand the function of Kinesin-I in long distance transport and in MT organization, we focused on the function of this complex in regulating *Drosophila* bristle development. Bristle cells represent the prominent visible component of the peripheral nervous system and cover much of the adult epidermis. The largest of these mechanosensory bristles (macrochaetes) are single cells featuring 250–400 μm-long extensions. Interestingly, it has been shown that mutations in *Drosophila Khc* affect bristle morphology (Brendza et al., 2000), with the major effect being seen in the upper part of the bristle, which exhibits flattened, flared, or twisted tips. In contrast, the cuticle layers of null bristles were quite thin, a property that is more pronounced at the tips of bristles than at their bases, suggesting that Khc plays a role in transporting essential precursors for membrane construction (Brendza et al., 2000). Still, the molecular mechanism by which Khc regulates the elongation and morphology of the highly polarized bristle cells remains unknown. It has been shown that MTs are essential for maintaining the highly biased axial growth of the *Drosophila* bristle (Fei et al., 2002) and for mediating protein and membrane transport (Fei et al., 2002; Tilney et al., 2000). It has also been suggested that bristle MTs are highly stable, forming at the start of the elongation and then extending along the shaft as the cell elongates (Tilney et al., 2000). Recent work from our group has demonstrated that the bristle shaft contains two populations of MTs (Bitan et al., 2010a, 2010b, 2012). The first comprises MTs that are stable and uniformly oriented with minus ends pointed toward the bristle tip, and are believed to serve as a polarized track for proper organelle and protein distribution. The second MT population is dynamic and presents mixed polarity. It is thought that the group contributes to proper axial growth and the establishment of bristle polarity (Bitan et al., 2012). As such, this unique MT organization in a highly polarized cell makes the bristle an ideal model for understanding the role of MT-associated motor proteins in long distance transport and MT organization.

In this study, we found that the Kinesin-I complex is required for bristle development, mainly by affecting bristle tip morphology. Loss of stable of MTs at the bristle tip, along with defects in orienting dynamic MTs in *Khc* and *Klc* mutant flies, explains the morphological defects seen in the bristles of adult flies. We also revealed that Dhc64C is the primary motor protein responsible for both anterograde and retrograde mitochondrial transport in the bristle and that Khc but not Klc serves as an opposing motor for Dhc64C.

## RESULTS

### Identification of Khc and Klc mutant lines

**Fig. 1.**
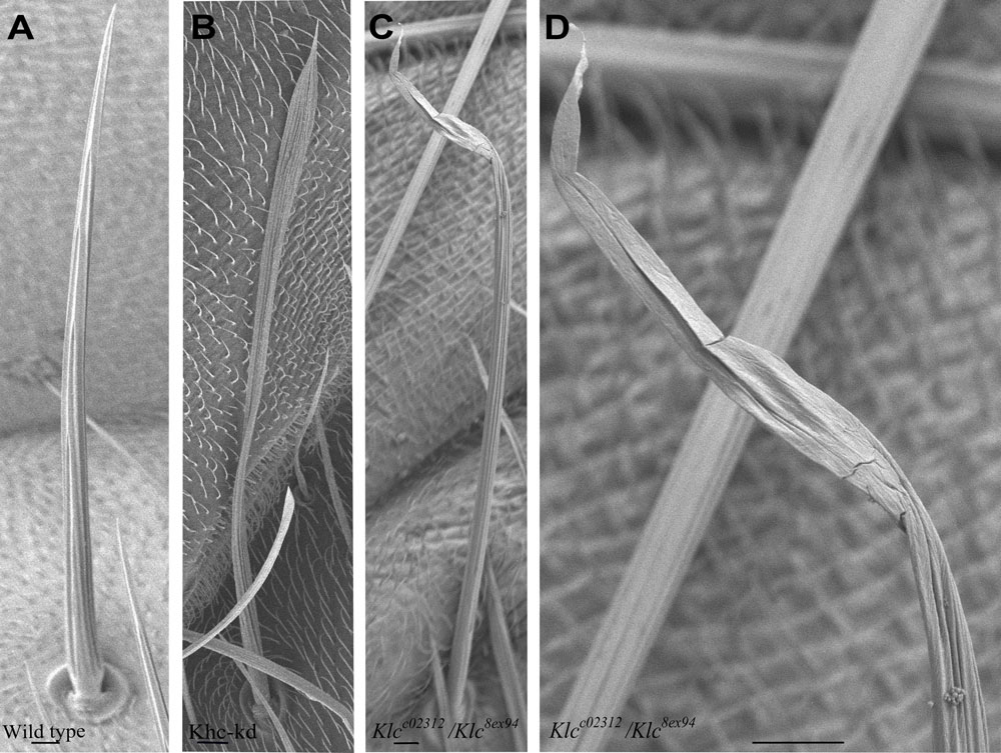
***Khc* and *Klc* are required for bristle development.** Scanning electron micrographs of wild-type (A), *Khc-RNAi; neur-Gal4* (B) and *Klc^c02312^*/*Klc^8ex94^* (C) bristles. (D) Enlargement of tip region from *Klc^c02312^*/*Klc^8ex94^* in C. *Khc-kd* and *Klc* mutants bristle fail to taper relative to their wild type counterparts. The wider and thinner tip region of the mutant bristles has abnormally organized surface grooves. Scale bars: 10 µm.

To investigate the role of *Khc* in bristle development, we searched for *Khc* alleles (Djagaeva et al., 2012) that give rise to at least pharate adults that would allow us to investigate the role of Khc in bristle development. However, all available alleles that determined to be either homozygous or trans-heterozygous were lethal at larval stages. Thus, to study the role of kinesin in bristle development, we used conditional RNA interference (the UAS-GAL4 system) to specifically knock down *Khc* mRNA levels in the bristles. Previous use of lines developed for RNAi-mediated knock down *Khc* mRNA levels (*Khc-kd*) (Mummery-Widmer et al., 2009; Schmidt et al., 2012) revealed that defects in bristle morphology leads to similar defects as seen in in *Khc* loss of function flies (Fig. 1), with effects being mostly detected in the upper part of the bristle (Brendza et al., 2000).

Next, we searched for novel *Klc* alleles that would allow us to examine the role of this gene in bristle development. The null *Klc* allele *Klc^8ex94^,* which deletes most of the *Klc* transcription unit, is homozygous lethal at the larval stage (Gindhart et al., 1998). Thus, to study the function of *Klc* in bristle development, we used an available known transposon insertion allele associated with the *Klc* gene, *Klc^c02312^*, and tested whether this allele was able to complement the *Klc^8ex9^*^4^ mutant phenotype. We found that the trans-heterozygotes were lethal and flies reached the stage of pharate adulthood but died before eclosion, allowing for the tracking of bristle development. We also found that homozygous *Klc^c02312^* flies died as pharate adults. Closer examination of these pharate adults by scanning electron microscopy (SEM) revealed that they presented defects in their bristle morphology (Fig. 1). Throughout the paper, we will refer to *Klc^c02312^/Klc^8ex94^* trans-hetrozygous flies as *Klc* mutants, unless otherwise mentioned. To confirm that lethality and the observed defects in *Klc^c02312^* bristles were due to P-element insertions, we excised these moieties from the germ line. We found that the revertants were viable and did not show bristle defects. Moreover, crossing *Klc^8ex94^* flies with the revertants revealed the resulting trans-heterozygous individuals to be viable, with no bristle defects.

### Defects in bristle development in Kinesin-1 mutant flies

To better characterize morphological defects in *Khc-kd* and *Klc* mutant bristles, the external cuticular structure of macrochaetes was examined by SEM. Wild-type bristle cells assume a prolonged cone-like shape with a wide base and sharp tip area (Fig. 1A). The external bristle surface has a characteristic grooved morphology with precisely positioned valleys and ridges running in a parallel manner from the base to the tip of the bristle. We found that in both the *Khc-kd* (95%, *n*=27 bristle) and the *Klc* (98%, *n*=37 bristle) mutants, the overall shape of the shaft was strongly affected, as manifested by a change in diameter along the length of the bristle. In both *Khc-kd* (Fig. 1B) and *Klc* mutant (Fig. 1C,D) flies, the bristle failed to taper towards the tip. Moreover, the upper part of the bristle was thinner and flat, with abnormally organized surface grooves and smaller (Fig. 1C,D) or completely missing (Fig. 1D) ridges. Next, we examined whether *Khc* knock down in the bristle affected bristle length. No significant differences bristle length in *Khc*-*kd* (327±33; *n*=20), *Klc* mutants (328±35; *n*=10) and wild-type flies (313±42; *n*=12) were noted. The striking similarity of the bristle defects in *Khc-kd* and *Klc* mutant flies suggests that the Khc and Klc proteins function together during bristle development. Accordingly, we next considered how Kinesin-1 controls bristle development.

### Defects in MT distribution are seen in the upper part of Kinesin-1 mutant bristles

**Fig. 2.**
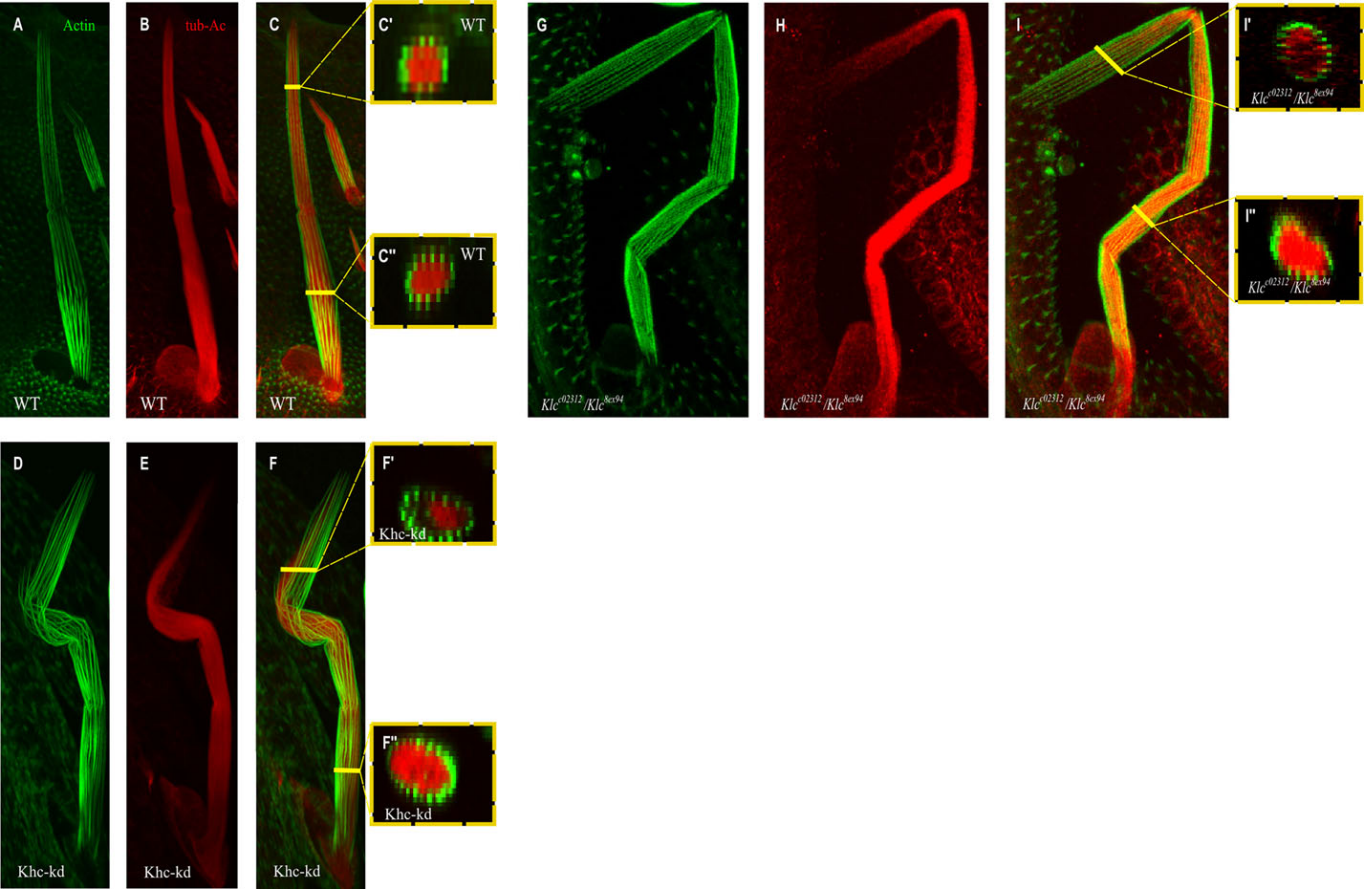
***Khc-kd* and *Klc* mutants affect microtubule distribution at the bristle upper part.** Confocal projections of bristles from wild type (A-C), *Khc-RNAi; neur-Gal4* (D-F) and *Klc^c02312^*/*Klc^8ex94^* (G-I) pupae stained with Oregon green-phalloidin (green) and with anti-acetylated α-tubulin antibodies (red). Digital cross-section as marked in yellow line of wild type (C′,C″), *Khc-kd* (F′,F″) and *Klc^c02312^*/*Klc^8ex94^* (I′,I″) demonstrate gradual decrease in stable MTs density towards the upper part of *Khc-kd* and *Klc* mutant bristle.

*Drosophila* bristle cell elongation during metamorphosis takes 16-18 h (Tilney et al., 2004). Since *Khc-kd* and *Klc* mutant bristles exhibited aberrant morphology that appeared to be cytoskeleton-related, cytoskeleton organization in these flies was analyzed during pupal development. In contrast to wild type (Fig. 2A-C), we noted that the upper part of the developing *Khc-kd* (Fig. 2D-F, 90% of 14 bristles) and *Klc* mutant (Fig. 2G-I, 95% of 19 bristles) bristle failed to taper towards the tip. Phalloidin staining revealed normal actin bundle organization in the *Khc-kd* (Fig. 2D) and in *Klc* mutants (Fig. 2G) bristles. Next, we investigated the organization of MTs in *Khc-kd* fly bristles. Normally, dense MT arrays fill the entire bristle shaft from base to tip, with the MT filaments running longitudinally as short overlapping fragments (Tilney et al., 1995). It was previously shown that the MT array in the bristle is composed of stable and dynamic MT populations (Bitan et al., 2012). To analyze the organization of the stable MT population, developing bristles were stained with anti-acetylated α-tubulin antibodies. In wild-type bristles, MTs were abundant along the entire shaft length (Fig. 2B,C,C′,C″). In *Khc-kd* (Fig. 2E,F) and *Klc* mutant (Fig. 2H,I) bristles, MTs were evenly distributed throughout the bristle shaft (Fig. 2F′,I′) but were reduced from most of the abnormally wide upper region of the cell (Fig. 2F″,I″).

**Fig. 3.**
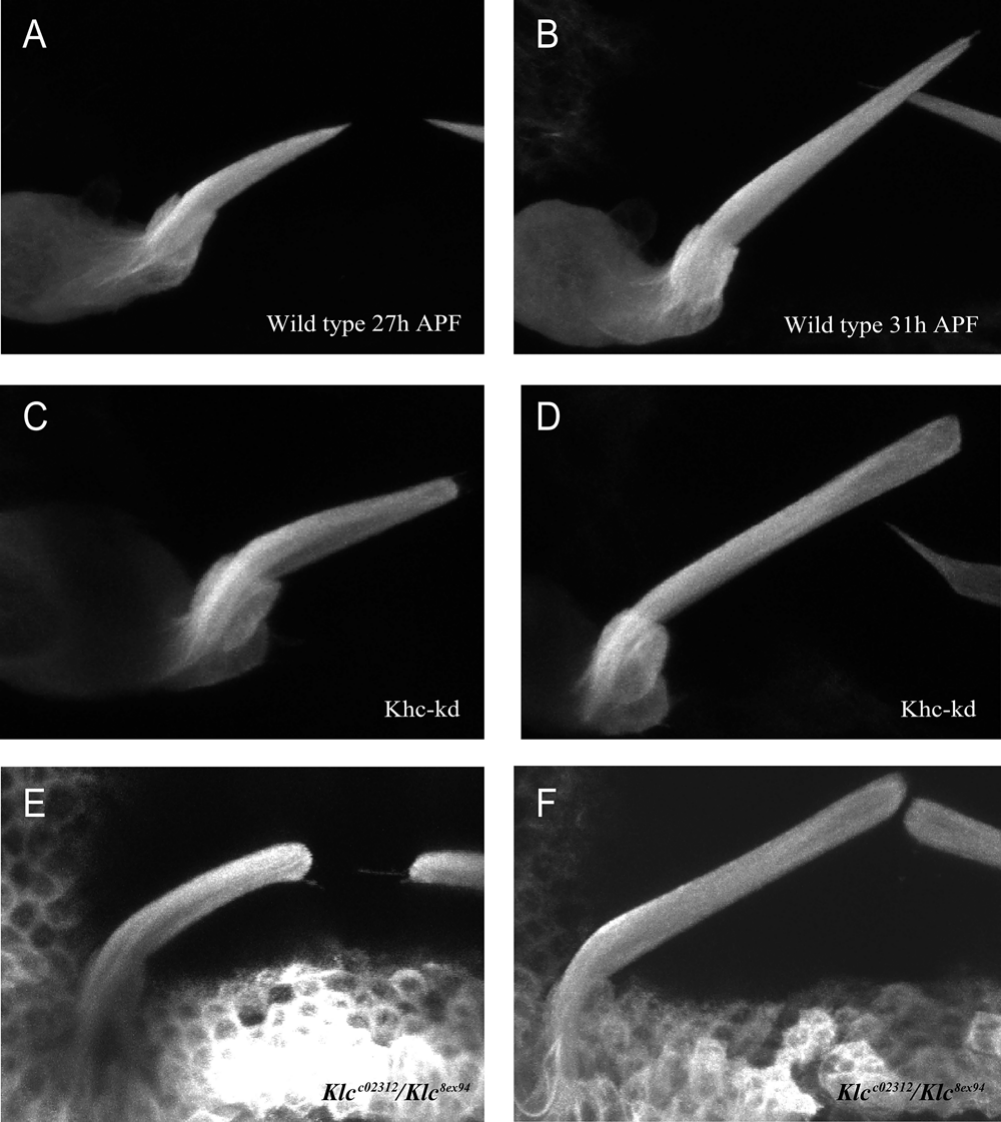
***Khc-kd* and *Klc* mutants fail to taper at the beginning of bristle development.** Confocal projections of bristles from wild type (A,B), *Khc-RNAi; neur-Gal4* (C,D) and *Klc^c02312^*/*Klc^8ex94^* (E,F) pupae expressing end binding protein 1 (EB1), a MT-plus end protein, attached to GFP, during bristle elongation. Number of hours after puparium formation =27 h in A,C,E and 31 h in B,D,F. Wild-type (*Neur-Gal4 EB1*) bristles are sharpened from the beginning of creation (A,B), whereas pUAS *Khc* *RNAi;* *Neur-Gal4 EB1* (C,D) and *Sca-Gal4 EB1*; *Klc^c02312^*/*Klc^8ex94^* (E,F) bristles are wide from the beginning and become wider as the bristle elongates.

To determine at which stage of bristle development the cell failed to taper, we tracked bristle elongation in cells expressing a MT plus end-binding protein termed end-binding protein 1 (EB1) fused to GFP (Bitan et al., 2012) to mark the bristle shaft (Movie 1 is representative results of 5 elongating tracking from each genotype). We found that whereas in early stages of the elongation process wild type developing bristles presented tapered tips (Fig. 3A,B; Movie 1), the tips were blunt-shaped from the beginning of the elongation process and became even wider as the bristle elongated in *Khc-kd* (Fig. 3C,D; Movie 1) and *Klc* mutant (Fig. 3E,F; Movie 1) bristles. These results thus suggest that Khc, together with Klc, is required for the characteristic tapered structure of the bristle tip.

### Bristle mitochondrial transport requires Khc and Dhc64C

**Fig. 4.**
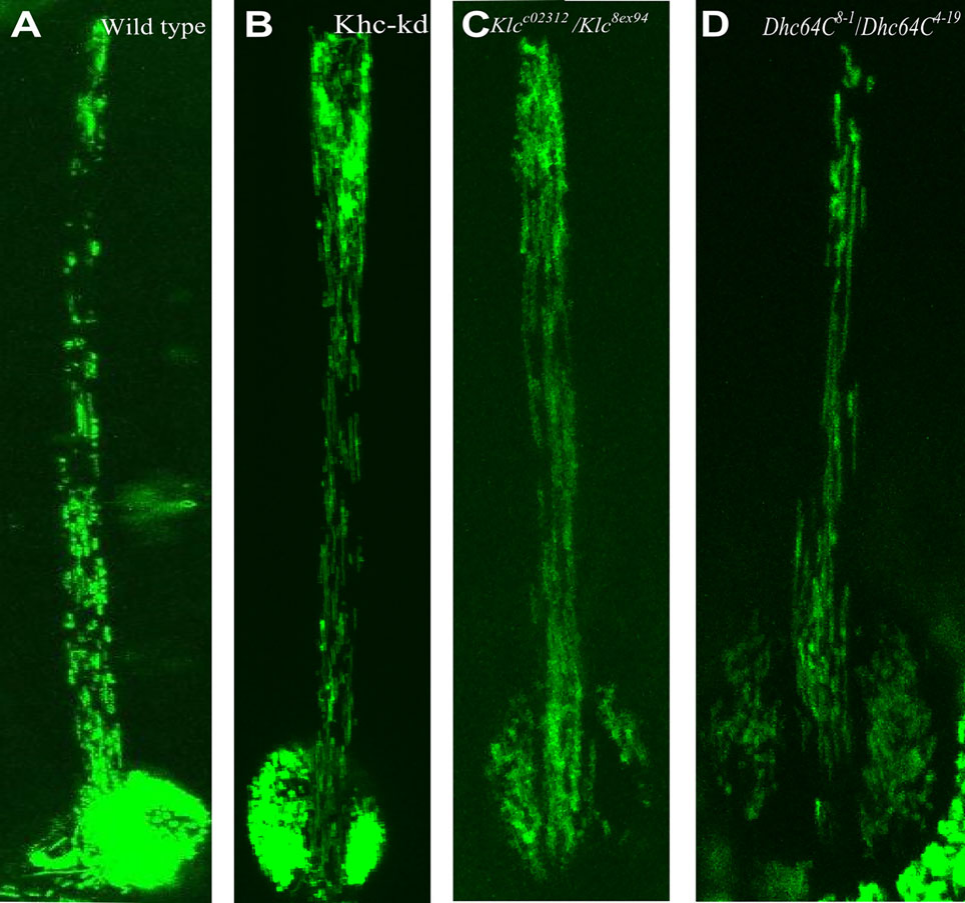
**Bristle mitochondria distribution shows no detectable defects in *Khc-kd*, *Klc* and *Dhc64C* mutants.** Confocal projections of bristles expressing mitochondria marker, UAS-GFP-Mito from wild type (A), *Khc-RNAi; neur-Gal4* (B), *Klc^c02312^*/*Klc^8ex94^* (C) and *Dhc64C^8-10^*/*Dhc64C^4-19^* (D). The mitochondria localization pattern shows a similar wild-type distribution in *Khc-kd*, *Klc* and *Dhc64C* mutants.

Previous studies have shown that mutations in *Khc* caused substantial accumulation of organelles, including mitochondria, in neurons, leading to axonal swelling. This phenomenon is often called ‘organelle jam’ (reviewed by Pilling et al., 2006). It was suggested that such axonal swelling is not a result of simple physical transport blockage but would instead reflect sites of autophagocytosis of senescent mitochondria (Pilling et al., 2006). We thus reasoned that swelling of the bristle, along with the absence of MTs in the upper part of the *Khc-kd* and *Klc* mutant bristle, might be due to jammed trafficking of organelles, such as the mitochondria. To test mitochondrial distribution in mutant fly bristles, we addressed transgenic flies containing Gal4-responsive mitochondria-targeted GFP (Mito-GFP). We found that in the wild type, mitochondria were distributed throughout the bristle shaft (Fig. 4A). A similar distribution of mitochondria was detected in *Khc-kd* (Fig. 4B) and *Klc* mutant (Fig. 4C) bristles, with no visible accumulation at the swollen upper part of the bristle (Fig. 4B,C). We found that also in *Dhc64C* mutants (*Dhc64C^8-1^*/*Dhc64C^4-19^*), mitochondria were distributed throughout the bristle shaft (Fig. 4D), similarly to wild type.

Although we were not able to detect any obvious defects in mitochondrial distribution in *Khc-kd* (Fig. 4B), *Klc* (Fig. 4C) and *Dhc64C* (Fig. 4D) mutants, we decided to check whether these mutants affected mitochondrial movement within the bristle, as was previously described in neurons (Pilling et al., 2006). It was further shown in the neuron that axonal transport of mitochondria requires Milton but not Klc as an adaptor protein (Glater et al., 2006). We found that in wild-type bristles, mitochondrial movement is highly dynamic, with approximately 95% of mitochondria moved vigorously in one direction and the rest being either stationary or displayed short salutatory movements with no explicit direction (Movie 2). In our analysis, only the moving mitochondria were tracked. The first parameter tested was the direction of movement. Each movement was defined as anterograde (moving toward the bristle tip) and retrograde (moving toward the bristle base). Time lapse confocal microscopy showed highly biased directionality, with 74%±5% of the mitochondria moving anterograde (Table 1). Next, we measured the velocity of the moving mitochondria and found that the range of velocities for both anterograde and retrograde was broad (0.26-5.10 μm/s anterograde, 0.38-4.4 μm/s retrograde; Movie 2). The net velocity of anterograde movement was 2.09±0.08 μm/s, while that of retrograde movement was 2.03±0.18 μm/s (Table 1).

**Table 1.**
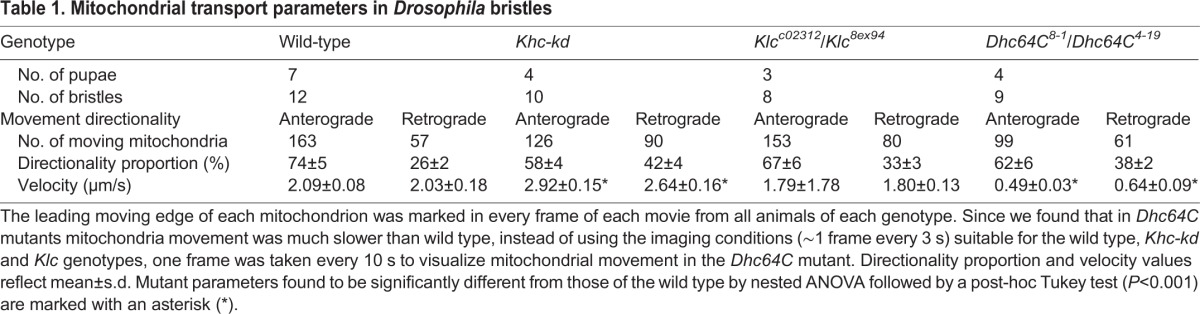
**Mitochondrial transport parameters in *Drosophila* bristles**

Since, stable bristle MTs are organized minus-end-out, we reasoned that that the only cytoplasmic Dhc64C will be the primary motor for anterograde mitochondrial movement. First, we showed that in *Dhc64* mutants 62±6% of the mitochondria moved in the anterograde direction, which showed no significant difference as compared to wild-type bristle (Table 1, Movie 2). However, we found that the net velocity of moving mitochondria was significantly reduced in both directions, with the net velocity of anterograde mitochondria being 0.49±0.03 μm/s (*P*<0.001) and the net velocity of retrograde mitochondria being 0.64±0.09 μm/s (*P*<0.001) (Table 1).

Next, we analyzed mitochondrial movement in *Khc-kd* bristles and found no significant change in the anterograde proportion of mitochondrial transport (Movie 2). We also found that the net velocities of both the anterograde (2.92±0.15, *P*<0.001) and retrograde (2.64±0.16, *P*<0.001) movements were significantly higher, as compared to wild-type mitochondrial movement. As for Klc, both the proportion of mitochondria that moved in anterograde direction (67%±6%) and the net velocity (anterograde 1.8±0.13 μm/s, retrograde 1.79±1.78 μm/s; Movie 2) were not significantly different from what was measured in the wild-type bristle.

### MTs are reduced in the upper part of *Khc-kd* and *Klc* mutant bristles

**Fig. 5.**
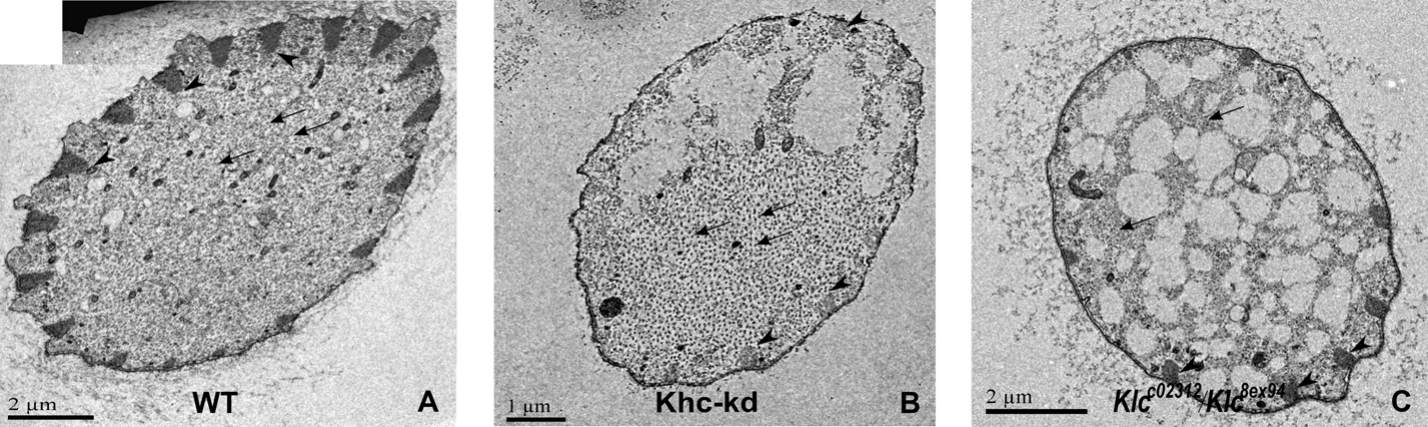
**Stable MTs are reduced in the upper part of *Khc-kd* and *Klc* mutants bristle.** (A-C) TEM micrographs of cross-sections through bristles of wild-type (A), *Khc-RNAi; neur-Gal4* (B) and *Klc^c02312^*/*Klc^8ex94^* (C) flies. Areas lacking MTs and other organelles were found in *Khc-RNAi; neur-Gal4* (B) and in *Klc^c02312^/Klc^8ex94^* (C) flies. Arrowheads point to the actin bundles and arrows indicate microtubules.

Our results indicate that the absence of MTs in the upper part of Kinesin-1 mutant flies is not due to physical barrier created by ‘jammed’ organelles. Thus, we decided to use transmission electron microscopy (TEM) of thin sections to visualize MT distribution within the bristles of *Khc-kd* and *Klc* mutant flies. We noticed that whereas in wild-type bristles MTs are found throughout the entire bristle shaft (Fig. 5A), *Khc-kd* (Fig. 5B, 93% of 4 examined bristles) and *Klc* (Fig. 5C, 95% of 6 examined bristles) mutant bristles contained an area that lacked MTs or any organelles, demonstrating that Khc and Klc are required for MT distribution, at least in the upper part of the bristle.

In *Drosophila* oocytes, both kinesin and cytoplasmic dynein concentrate at the posterior pole (Clark et al., 1994; Li et al., 1994). However, in *Khc* mutants, dynein heavy chain (Dhc) staining showed little or no posterior localization. Rather, Dhc strongly accumulated at the anterior end of the oocyte. The shift of dynein to the anterior pole in *Khc* mutants suggests that kinesin is responsible for moving cytoplasmic dynein away from MT minus ends at the anterior end and thus, towards the posterior pole (Brendza et al., 2002). Previous research has shown that Dhc tends to accumulate in the tips of wild-type bristles (Bitan et al., 2012). Considering these results, we were interested in assessing whether mutations in *Khc* and *Klc* would affect Dhc localization. Therefore, we determined the localization of Dhc in the bristle using transgenic flies expressing HA-tagged Dhc under control of the endogenous promoter (Silvanovich et al., 2003). We found that whereas in the wild type (Fig. S1A,B) Dhc tightly accumulated at the bristle tip, in both *Khc*-*kd* (Fig. S1C,D) and *Klc* mutant bristles (Fig. S1E,F), Dhc was still localized to the tip, albeit more diffusely than in the wild-type bristle.

### Kinesin-1 is required for dynamic MT orientation at the bristle tip

**Fig. 6.**
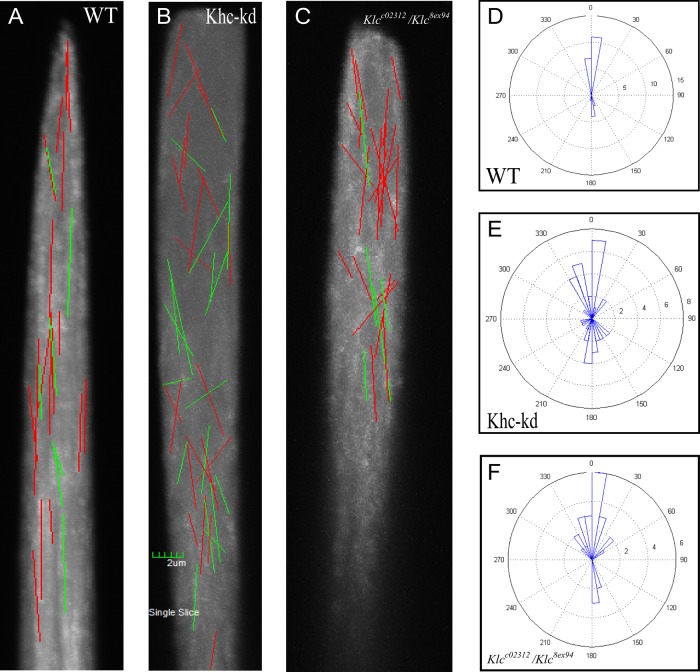
***Khc-kd* and *Klc* are required for orienting dynamic MTs at the bristle tip.** (A-C) Tracking GFP-EB1 in bristles in wild-type (A), *Khc-RNAi; neur-Gal4* (B), and *Klc^c02312^*/*Klc^8ex94^* (C) flies. Red lines indicate tip-directed tracks and green lines indicate base-directed tracks. (D-F) Angular distributions of the direction of GFP-EB1 comets in wild type (D), *Khc-RNAi; neur-Gal4* (E), and *Klc^c02312^*/*Klc^8ex94^* (F) flies.

The intriguing finding that stable MTs are reduced in the upper part of the bristle tip led us to track dynamic MT polymerization within the elongating shaft. Using the UAS-GAL4 expression system, we expressed GFP-EB1 in bristles and tracked EB1 movement after initiation of shaft elongation in pupae aged 36-42 h, after puparium formation (APF). We tested the angle of deviation from the central growth axis. As such, angles of EB1 movement with regard to the shaft central axis were measured and the mean of the angles was calculated in the bristles of wild-type and in *Khc-kd* and *Klc* mutant flies and compared using a nested ANOVA test (individual bristles nested according to genotype). In the wild-type bristle, EB1 movements in the upper part of the bristle ran almost parallel to the axis of the shaft, with the mean of central axis deviation angles being 7.3±8.4 (*n*=271; Fig. 6A,D, Movie 3). However, in both *Khc-kd* (16.09±13.99, *n*=234; Fig. 6B,E, Movie 3) and *Klc* (16.39±13.60; *n*=145; Fig. 6C,F, Movie 3) mutant bristles, highly significant deviations (*F*_12,635_=56.09, *P*<0.0001) in angle distribution were recorded. Moreover, no difference in the angles of EB1 movement between the two mutants was found. These results demonstrate that in both *Khc* and *Klc* mutants, dynamic MT array spatial orientation is highly disrupted. The striking similarity between the angles of deviation in both mutants suggests that Khc and Klc act together in directing the MTs, at least in the tip region of the bristle.

### GFP-EB1 fails to concentrate at the bristle tip in Kinesin-1 mutants

**Fig. 7.**
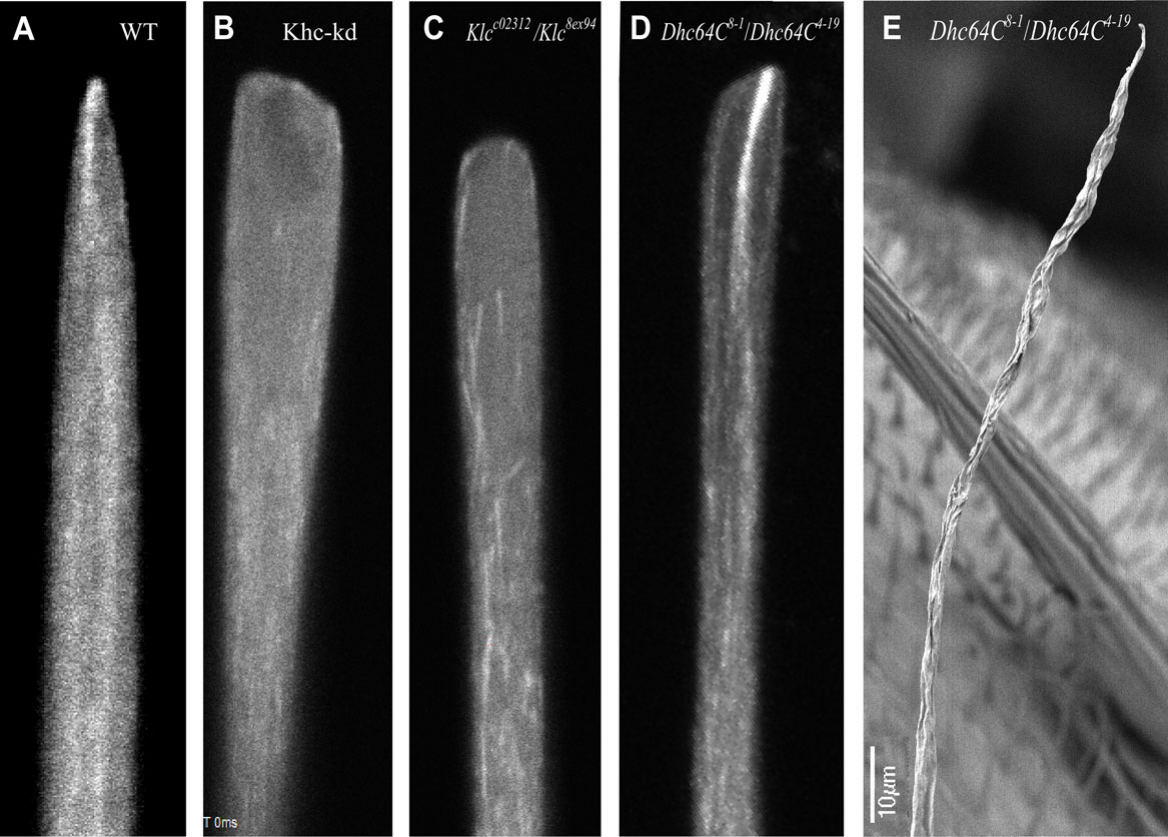
**GFP-EB1 fails to concentrate as foci at the bristle tip in *Khc-kd* and *Klc* mutants.** (A-D) Confocal projections of bristles from wild type (A), *Khc-RNAi; neur-Gal4* (B) and *Klc^c02312^*/*Klc^8ex94^* (C) and *Dhc64C^8-10^*/*Dhc64C^4-19^* (D) mutants expressing GFP-EB1. (E) Scanning electron micrographs of upper part of bristle from *Dhc64C^8-10^*/*Dhc64C^4-19^* mutants. GFP-EB1 concentrates as foci at the tip of the bristle both in wild type and in *Dhc64C^8-10^*/*Dhc64C^4-19^* but not in *Khc* and *Klc* mutants. *Dhc64C* mutants bristle tip is thin and twisted.

To better understand the mechanism by which Khc and Klc might orientate dynamic MTs, we examined the pattern of GFP-EB1 distribution at the bristle tip. In the wild type, GFP-EB1 was found to accumulate as a distinct focus at the sharp edge of the bristle tip (Fig. 7A). In comparison, in both *Khc-kd* (Fig. 7B) and *Klc* (Fig. 7C) mutant bristles, GFP-EB1 failed to accumulate as a single focus in the swollen tip. Two explanations for the fact that GFP-EB1 failed to concentrate at the mutant bristle tip are possible. In the first, the appearance of blunt tip morphology is responsible for the failure of EB1 to concentrate as a single focus. Alternatively, Khc and Klc might be directly involved in orchestrating the orientation of the dynamic MTs to form the EB1 focus at the growth edge; perturbation of *Khc* or *Klc* would thus affect bristle tip shape. To distinguish between these possibilities, we searched for other mutants which present a wide tip yet maintain a single EB1 focus. We found that during the cell elongation, the *Dhc64C* mutant tip failed to taper (Fig. 7D), although by the end of bristle development, the upper part (specifically about one-third of the length of this region) was extremely thin and twisted (Fig. 7E), and lacked the characteristic grooved surface. Closer examination of EB1 organization in the *Dhc64C* mutant tip revealed that EB1 had concentrated at the highly defined point at the tip of the bristle. These results show that similarly to *Khc-kd* and *Klc* mutants, *Dhc64C* bristles fail to taper during the elongation. However, in contrast to the *Khc-kd* and *Klc* mutants, EB1 accumulated as a focus at the bristle tip. Furthermore, the swollen tip containing a concentrated EB1 focus in the *Dhc64C* mutants resulted in a bristle morphology (Fig. 7D), which differed from *Khc-kd* and *Klc* mutants. Hence, the loss of MT foci is the primary reason for the blunt tip morphology observed in *Khc* and *Klc* mutant bristles, with the Kinesin-1 complex being responsible for establishing and maintaining EB1 foci in developing bristle tips.

## DISCUSSION

### The Kinesin-I complex is required for proper bristle development

Khc and Klc work together to mediate Kinesin-1 complex function. Yet, it has been reported that not all Khc functions are dependent on Klc. In *Drosophila*, mutations in both Khc and Klc result in similar neuronal defects that lead to axonal swelling and progressive posterior paralysis (Gindhart et al., 1998; Hurd and Saxton, 1996), suggesting that Khc and Klc function together by affecting multiple pathways of axonal transport. On the other hand, Khc-dependent axonal mitochondrial transport requires Milton instead of Klc as the adaptor protein (Glater et al., 2006). Moreover, in other *Drosophila* tissues, Khc is essential for mRNA localization, oocyte streaming and MT sliding, independently of Klc (Jolly et al., 2010; Lu et al., 2013; Palacios and St Johnston, 2002). In the present study, we found that Khc, together with Klc, is required for the establishment of proper bristle morphology. We showed that *Khc* and *Klc* mutants fail to taper towards the tip and that the tip region is mis-organized in these mutants. We found that the Khc but not Klc is required for mitochondrial movement in the developing bristle as opposing force for the primary motor protein Dhc64C. We further demonstrated that stable MTs were reduced at the bristle tip in Kinesin-I mutants. We showed that Khc and Klc are required for orienting dynamic MTs in the tip region by maintaining an EB1 focus at the sharpened growth edge of the cell.

Experimental evidence provides possible explanations for the role of Klc in Kinesin-1 complex activity. As *Khc* and *Klc* mutants share identical phenotypes, at least in terms of axonal development (Gindhart et al., 1998; Hurd and Saxton, 1996; Saxton et al., 1991) and, as revealed here, in bristle morphology, it was suggested that Klc positively controls Khc function. Specifically, Klc may inhibit motor activity in the absence of cargo, yet would activate such activity upon cargo binding (Kamal and Goldstein, 2002). Alternatively, it was suggested that Klc couples the heavy chain to its cargo (Kamal and Goldstein, 2002). However, it was shown that this activity is dependent on the other cargo adaptors proteins, such as Syd in *Drosophila* or its mammalian homologues JIP3/JSAP1, JIP1, amyloid precursor protein (APP) and related APP-like proteins (Bowman et al., 2000; Hammond et al., 2008; Kamal et al., 2000; Loiseau et al., 2010; Torroja et al., 1999; Verhey et al., 2001; Zheng et al., 1998). Knocking down *Drosophila syd* and *APP* transcript levels had no effect on bristle morphology development (Mummery-Widmer et al., 2009), suggesting that in contrast to what occurs in neurons, the Kinesin-I complex cargo transport mechanism does not require these known adaptor proteins during bristle development. Nonetheless, further analysis will be required to understand the molecular mechanism by which Klc regulates Khc activity during neuron and bristle development.

### Kinesin-I and mitochondrial transport during bristle development

The fact that Khc is required for axonal mitochondria transport (Glater et al., 2006; Hurd and Saxton, 1996; Tanaka et al., 1998) led us to assess mitochondrial distribution in *Khc* mutant bristles. We demonstrated that whereas *Dhc64C* significantly inhibited both mitochondrial anterograde and retrograde net velocities, mutation in *Khc* significantly enhanced these values. Moreover, we found that *Klc* had no effect on mitochondrial transport in the bristle. The fact that Klc is not required for Khc-dependent mitochondrial transport in the bristle is not surprising, since it was shown that Milton but not Klc serves as a mitochondrial adaptor protein (Glater et al., 2006). Since *Khc* and *Klc* mutants have the same bristle morphology defects, yet serve different roles in mitochondrial transport, it may be that the defects in bristle morphology are not due to the defects in mitochondrial transport.

How then is mitochondrial transport regulated during bristle development? It was shown that *Drosophila* axon MTs are organized uniformly with the plus end-out (Stone et al., 2008), and that Khc and Dhc64C are the primary motors for anterograde and retrograde mitochondrial transport (Pilling et al., 2006). Moreover, it was suggested that Khc is critical for retrograde transport by dynein, yet there is no evidence that force production by the two opposing motors is competitive (Pilling et al., 2006). Bristle MTs are organized minus end-out, suggesting that the major anterograde motor protein should be dynein, and indeed, we found a significant decrease in the velocity of anterograde mitochondria transport in *Dhc64C* mutants. To our surprise, mitochondrial movement in the retrograde direction was also much slower, as compared to wild-type. In addition, mutation in *Khc* enhanced the velocities of mitochondrial anterograde and retrograde transport. Given these results, we suggest the following model for mitochondrial transport in the bristle. We propose that *Dhc* is required for both retrograde and anterograde transport, since a majority (97±2.5%) but not all bristle MTs are organized with their minus ends towards the tip, such that any plus end-out oriented MT could serve as tracks for the low level of dynein-dependent retrograde mitochondrial transport. The fact that mutations in *Khc* enhanced mitochondrial movement velocity in both directions suggests that *Khc* serves as an opposing but not alternating force for dynein-dependent mitochondrial transport. To conclude, we report for the first time a case in which dynein serves as primary motor protein for mitochondrial transport, with Khc acting as the opposing motor.

### Khc and MT organization and function

We found that mutations in *Khc* and *Klc* affect the external structure of the bristle, with the main defect observed at the bristle tip region being the appearance of a blunt and flat structure presenting disrupted surface grooves. Elongating bristles failed to taper and displayed an abnormally blunted and wide tip region in early developmental stages. Closer examination using both confocal and electron microscopy revealed that the upper part of the bristle contained areas that lack stable MTs. We revealed that at the bristle tip, mutations in *Khc* and *Klc* alter the direction of MT polymerization.

What is the mechanism by which Kinesin-1 affects stable MT organization and dynamic MT orientation, leading to the defects found in bristle at the adult thorax when *Khc* or *Klc* are mutated? One explanation is that Kinesin-1 is required for the early distribution of the MT network, such that a lack of stable MTs affects the growth direction of dynamic MTs. It was suggested that 75% of the stable MTs form early in bristle development, and that these then slide out during bristle shaft elongation (Tilney et al., 2000). As stable MTs in the bristle are organized minus end-out, it would thus appear that plus end motor protein push the MTs during cell elongation. Indeed, it was shown that Khc is required for MT sliding in mammalian, *Drosophila* and *C. elegans* cells (Lu et al., 2013; Metzger et al., 2012; Yan et al., 2013). Khc-mediated MT sliding activity is Klc-independent (Jolly et al., 2010; Lu et al., 2013), however, we showed that MTs were reduced in the bristle tip in *Klc* mutant flies. This implies that defects in the bristle tip MT organization are not due to MT sliding defects. A second explanation for the defects noted in the organization of stable and dynamic MTs at the tip of Kinesin-1 mutant bristles is that this complex primarily affects dynamic MTs, which then in turn affect the stable MT population. Support to this hypothesis is provided by the finding that the plant-specific kinesin KINID1 regulate the turnover of the MTs at the expansion zone of the moss *Physcomitrella patens* by stabilizing a single coherent focus of MTs and GFP-EB1 in the center of the zone (Hiwatashi et al., 2014). Indeed, we found that whereas in wild-type bristles GFP-EB1 is concentrated as a focus at the tip of the bristle, this focus is lost in Kinesin-1 mutants. Still, the absence of GFP-EB1 foci could be a result of primary defects in stable MT organization. To address this point, we sought other mutants that affect bristle tip morphology during development in a manner similar to Kinesin-1 mutants but which ultimately lead to different defects in the adult bristle. During our studies on the role of Dhc64C in bristle development, we found that as with Kinesin-1 mutants, the tip region failed to taper during bristle development, albeit differently from the Kinesin-1 mutants. In the adult bristle of *Dhc64C* mutants, the tip region was narrow and twisted, but still managed to maintain characteristic pointed tip. Closer examination of GFP-EB1 localization in the *Dhc64C* mutants revealed that GFP-EB1 is concentrated as a single coalescence of growing MT plus ends.

Our previous work has shown that bristle dynamic MTs are required for proper axial growth and the establishment of bristle polarity (Bitan et al., 2010a, 2010b). By manipulating MT dynamics following Klp10A RNAi down-regulation, we have shown that dynamic MTs can initiate new shaft extensions and thus, have the ability to direct bristle growth (Bitan et al., 2012). Taking this into consideration, along with our results presented here, a model in which dynamic MTs grow in a directional manner towards the tip in the wild-type bristle, as evident from the appearance of GFP-EB1 focalization at the bristle tip, is supported. Such directional polymerization of dynamic MTs ensures the tapering of the bristle towards the tip. In Kinesin-1 mutants, deviation of dynamic MTs from their growth axis generated a wide and flat tip. On other hand, in *Dhc64C* mutants, even though the tip region was wide during development, GFP-EB1 was found as a single coalescence towards the tip, ensuring tapering of the bristle tip, as in the adult bristle. In this case, the abnormally twisting of the upper part of the bristle results from other processes yet to be investigated.

The additional unanswered question is why the upper part of Kinesin-1 mutant bristles lacks stable MTs. This could be explained by a model proposing that a population of polarized dynamic MTs that are aligned along the cell axis is required for homeostasis length control in animal cells (Picone et al., 2010). In this model, the fate of MTs contacting the cell cortex depends on the contact angle, with wider angles leading to catastrophe by affecting MT stability. Thus, as in other cells expressing Kinesin-1 mutants, mis-oriented dynamic MTs fail to be stabilized. Alternatively, it is possible that the Kinesin-1 complex is required for MT cross-linking that would stabilize MTs in the upper part of the elongating bristle.

In summary, our results demonstrate a novel role of Kinesin-1 in mediating directional control of dynamic polarized MTs during polarized cell elongation using the *Drosophila* bristle as model tissue.

## MATERIALS AND METHODS

### *Drosophila* stocks

Oregon-R was used as wild-type control. The following mutant and transgenic flies were used: *Khc-IR* (Vienna *Drosophila* RNAi Center; V44337); Khc-TriP (Harvard Medical School, Boston, MA; 25898), *klc^c02312^* (*Drosophila* Stock Center, Bloomington, IN), *klc8ex94* (Gindhart et al., 1998), *Dhc64C^4-19^/TM6B, Dhc64C^8-1^/TM6B* (Gepner et al., 1996), *HA-Dhc64C* (Silvanovich et al., 2003), *pUAS GFP-EB1* (Satoh et al., 2008), *UAS-mito-GFP* (Pilling et al., 2006). Bristle expression was induced under the control of the *neur*-Gal4 driver.

### Developmental staging

Stages of all animals were determined from the point of puparium formation (Bainbridge et al., 1981). White prepupae were collected and placed on double-sided Scotch tape in a petri dish that was placed in a 25°C incubator, as previously described (Tilney et al., 2000). At the appropriate time of incubation (30 to 48 h APF, unless indicated otherwise), the Petri dish was removed and the pupae were dissected for live imaging, fixation or TEM preparation.

### Dissection of pupae

After removing the pupal case, the pupae were dissected as described elsewhere in detail (Tilney et al., 2000). The dissection procedure resulted in the isolation of thorax dorsal side tissue, which was then cleaned of interior organs and fat particles as described (Tilney et al., 2000). All procedures were conducted in phosphate-buffered saline (PBS). The isolated thoracic fillets were then subjected to antibody staining or TEM preparation.

### Bristle phalloidin and antibody staining

The procedure for fixation and staining was described previously (Guild et al., 2002). Briefly, the dissected thorax was transferred to 4% paraformaldehyde in PBS for 10 min, 4% paraformaldehyde containing 0.3% Triton X-100 in PBS for an additional 10 min, washed three times for 10 min in 0.3% Triton X-100 in PBS, and then blocked in 0.3% Triton X-100 containing 4% BSA and 0.1% NaN3 for 1 h. The sample was then incubated with primary antibodies in blocking solution overnight at 4°C, washed three times in 0.3% Triton X-100 in PBS and incubated with secondary antibodies in blocking solution overnight in the dark at 4°C. The sample was then washed three times for 10 min in 0.3% Triton X-100 in PBS and incubated with phalloidin in PBS overnight at 4°C, washed three times in 0.1% Triton X-100 in PBS and then placed on a slide and mounted in glycerol Citifluor (Ted Pella, Redding, CA, USA). Confocal images were taken on an Olympus FV1000 Laser-scanning confocal microscope and are presented here as *z*-projections of several optical sections that collectively cover the bristle diameter. The primary antibodies used were monoclonal mouse anti-acetylated tubulin (1:250; Sigma) and anti-Dhc (2C11-2, 1:50; Developmental Studies Hybridoma Bank) antibodies. The secondary antibodies used were Cy3-labeled goat anti-mouse (1:100; Jackson Immunoresearch) and Alexa Fluor 488-labeled goat anti-rabbit (1:100; Molecular Probes) antibodies. For actin staining, we used Oregon Green 488 phalloidin or Alexa Fluor 568 phalloidin (1:250; Molecular Probes).

### Scanning electron microscopy

Adult *Drosophila* flies were fixed and dehydrated by immersion in increasing concentrations of ethanol (25%, 50%, 75%, and 2×100%, each for 10 min). The samples were then completely dehydrated using increasing concentrations of hexamethyldisilazane (HMDS) in alcohol (50%, 75%, and 2×100%, each for 2 h), air-dried overnight, placed on stubs, coated with gold, and examined with a scanning electron microscope (JEOL model JSM-5610LV). Length measurements of adult bristles were made using Image J (version 1.40j).

### Organelle tracking and statistical analysis

Tracking of EB1 and mitochondria was manually performed using Image J software to measure the duration, start and end points of each EB1/mitochondria track. The data collected were then analyzed using Matlab R2010b (MathWorks). Annotated images were generated using the annotation function and Rose diagrams were generated using the Rose function. To test differences reflecting the deviation angle of EB1 movement, the angle of deviation from the central axis was calculated for each EB1 movement. A comparison of EB1 movement in wild-type and *Khc-kd* mutant flies was conducted using a nested ANOVA test in which individual bristles (random factor) were nested according to genotype (fixed factor). The angle of deviation of every EB1 movement was logarithmically transformed because of the skewed distribution. The following mitochondria transport parameters were defined: (1) the proportion of anterograde (towards the tip) and retrograde (towards the cell base) movement were compared between genotypes using one-way ANOVA followed by a post-hoc Tukey test; (2) to account for the differences in mitochondrial movement velocity along each track we used nested ANOVA with genotype as a fixed factor and mitochondrial velocity nested within genotype as a random factor, followed by a post-hoc Tukey test. Again, velocity was logarithmically transformed to correct for the skewed distribution of the data. All statistical analyses were conducted using STATISTICA version 12.0 software.
